# High prevalence of antibiotic use in a tertiary-care hospital in Sierra Leone: We need to handle antibiotics with care

**DOI:** 10.1017/ash.2023.242

**Published:** 2023-09-29

**Authors:** Ibrahim Franklyn Kamara, Bobson Fofanah, Bockarie Pharaj Sheriff, Victoria Katawera, Robert Musoke, Sulaiman Lakoh, Kadijatu Nabie Kamara, Joseph Sam Kanu

## Abstract

**Background:** Antimicrobial resistance (AMR) is a global public health concern that has the potential to reverse decades of progress aimed at decreasing morbidity and mortality attributed to infectious diseases. In 2019, ~5 million deaths were associated with AMR, of which 1.2 million were attributed to antibacterial-resistant infections. Healthcare facilities where antimicrobials are frequently used are high-risk settings for the selection and spread of resistant bacteria, and they further contribute to the increase in the burden of AMR. We have documented the prevalence and indication of antibiotic use in a tertiary-care referral hospital in Freetown, Sierra Leone. **Methods:** This point-prevalence survey was conducted at Connaught hospital, a tertiary-care hospital in Sierra Leone, in November 2021. The hospital offers a range of medical and surgical services through 25 units and has 16 wards with >300 beds. Data on patient-level antibiotic use, including indications for use, were extracted from medical records using WHO point-prevalence survey (PPS) forms that had been pretested and validated. Data collection was conducted in all the wards over a 10-day period by trained healthcare personnel. On the day of the survey, only the medical records of patients on admission before 8:00 a.m. on that day were included in the study. Data entry, cleaning, and analysis were conducted using the WHO PPS platform. Ethical approval was obtained. **Results:** In total, 87 patient records were included in the survey. Most (71%) were women, and the average age was 30.6 years. The prevalence of antibiotic use was 66%, and the average number of antibiotics prescribed to patients since admission was 2. The 5 most prescribed antibiotics were metronidazole, ceftriaxone, amoxicillin and clavulanic acid, ampicillin and cloxacillin, and azithromycin. The parenteral route of drug administration was the mainstay. The most frequent indications for antibiotic prescription were community-acquired infection and surgical prophylaxis. Blood-culture requests were not ordered before the initiation of antibiotic treatment. **Conclusions:** This study was the first study to be conducted in Connaught hospital using the WHO PPS methodology. The survey reports a high prevalence (60%) of antibiotic use, and most treatment was done empirically. This finding is contrary to the WHO recommendation of <30% antibiotic use. This high prevalence of antibiotic use has the potential to increase the burden of AMR in the country. Therefore, there is an urgent need to strengthen Connaught hospital’s antibiotic stewardship program.

**Figure:**
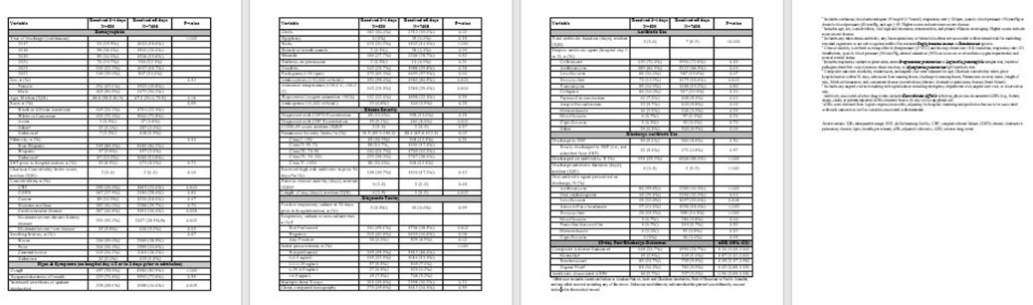

**Figure:**
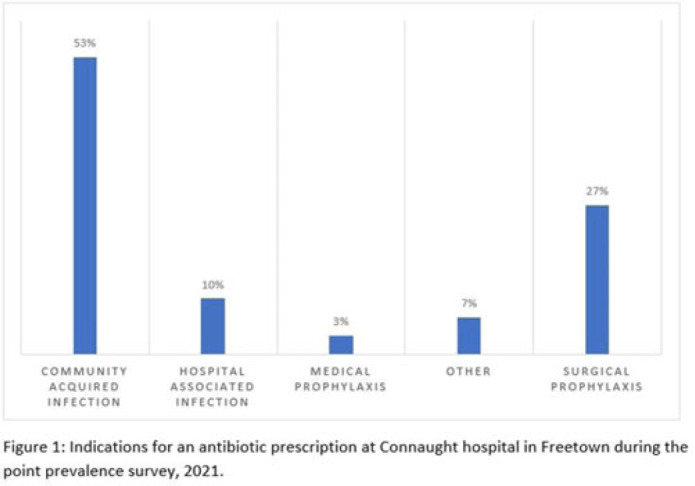

**Figure:**
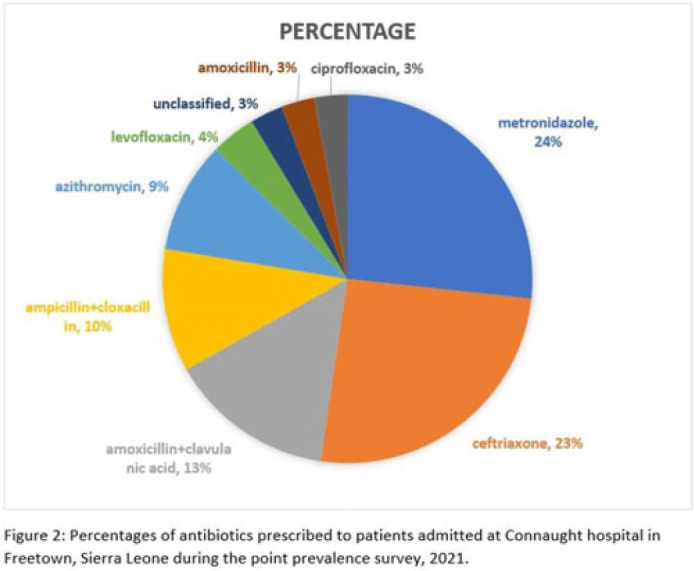

**Disclosures:** None

